# Preliminary clinical outcomes of head and neck squamous cell carcinoma treated with particle beam radiation therapy

**DOI:** 10.1002/cam4.5902

**Published:** 2023-04-11

**Authors:** Qingting Huang, Jiyi Hu, Weixu Hu, Jing Gao, Jing Yang, Xianxin Qiu, Haojiong Zhang, Jiade Jay Lu, Lin Kong

**Affiliations:** ^1^ Department of Radiation Oncology Shanghai Proton and Heavy Ion Center Shanghai 201321 China; ^2^ Department of Radiation Oncology Shanghai Proton and Heavy Ion Center Fudan University Cancer Hospital Shanghai 201321 China; ^3^ Shanghai Key Laboratory of Radiation Oncology (20dz2261000) Shanghai 201321 China; ^4^ Shanghai Engineering Research Center of Proton and Heavy Ion Radiation Therapy Shanghai 201321 China; ^5^ Department of Radiation Oncology Proton and Heavy Ion Center Heyou International Hospital Foshan China

**Keywords:** carbon ion, head and neck squamous cell carcinoma, proton, radiotherapy

## Abstract

**Purpose:**

Further improvement in clinical outcomes is needed for patients with head and neck squamous cell carcinoma (HNSCC), as there is typically a poor prognosis at diagnosis. This study aimed to report the preliminary therapeutic outcomes and side effects in patients with HNSCC receiving particle beam radiotherapy (PBRT), owing to the physical and biological advantages of this approach.

**Methods:**

We retrospectively analyzed 68 patients with newly diagnosed HNSCC who received PBRT at the Shanghai Proton and Heavy Ion Center (SPHIC) between August 2015 and December 2020. The Kaplan–Meier approach was used to determine overall survival (OS), disease‐specific survival (DSS), progression‐free survival (PFS), local recurrence‐free survival (LRFS), regional recurrence‐free survival (RRFS), and distant metastasis‐free survival (DMFS). Common Terminology Criteria for Adverse Events (CTCAE) 4.03 was also used to grade acute and late toxicities.

**Results:**

With a median follow‐up time of 24.5 months (range, 3–65), the 3‐year OS, DSS, PFS, LRFS, RRFS, and DMFS rates for the entire cohort were 79.0%, 84.7%, 67.9%, 83.5%, 83.3%, and 96.1%, respectively. Univariate and multivariate analyses showed that N category was a significant predictor of OS, PFS, and RRFS. In terms of acute toxicities, two patients demonstrated severe mucositis or dysphagia, and two patients also displayed a late toxicity of significant mucosal necrosis.

**Conclusion:**

These findings suggest that PBRT can provide patients with HNSCC with a promising therapeutic benefit and manageable toxicity. Prospective evaluation of clinical outcomes with PBRT for HNSCC is warranted, with an emphasis on clinical effectiveness as well as adverse effects and patient quality of life.

## Graphical abstract

Intensity‐modulated proton and/or carbon‐ion radiation therapy provided satisfactory therapeutic effectiveness in our head and neck squamous cell carcinoma patients with tolerable acute and late toxicities.

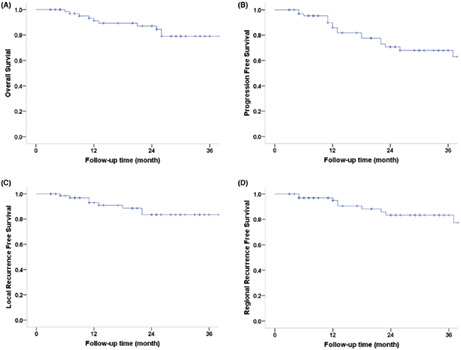

## 1 INTRODUCTION

With 890,000 new cases and 450,000 fatalities in 2018, head and neck squamous cell carcinoma (HNSCC) was the sixth most common malignancy worldwide. HNSCC is a diverse group of neoplasms derived from the mucosal epithelium in the oral cavity, oropharynx, hypopharynx, or larynx.^1^, ^2^, ^3^ Radiotherapy (RT) is considered the optimal standard of care for HNSCC, both for definitive or postoperative intent, with the advantage of preserving organs and their functions.^4^, ^5^, ^6^ However, 15%–50% of patients with HNSCC still have local or regional relapse after standard chemoradiotherapy regimens, suggesting that the current treatment modality of photon‐based chemoradiotherapy still has limitations in improving prognosis.^7^, ^8^ In addition, acute adverse effects, such as Grade 2 or higher mucositis, occur in approximately 60%–90% of HNSCC patients during RT.^9^, ^10^ Moreover, 68% of patients experienced voice problems after RT and presented long‐term symptoms.^11^ Hence, toxicities imposed by photon‐based RT may considerably lower the quality of life of patients with long‐term survival.^12^, ^13^

Particle beam RT (PBRT) is one of the most advanced RT techniques and exhibits significant physical and biological advantages. Previous studies have demonstrated the potential benefit of PBRT in the treatment of head and neck malignancy.^14^, ^15^, ^16^ A comparison of treatment planning between photons and protons or carbon ions for the treatment of HNSCC illustrated that proton‐ or carbon‐ion‐based planning produced better dose distribution compared to that of photon planning.^17^, ^18^ The use of proton and carbon ion RT in HNSCC has also shown a preliminary clinical outcome of reduced incidence of radiation‐induced toxicities such as weight loss or xerostomia.^19^, ^20^, ^21^ Furthermore, the biological advantages of particle beams have the potential to improve the survival of HNSCC patients in clinical settings.^15^, ^22^ In a study by Kitabatake et al. the 3‐year local control (LC) rate was 69% for elderly patients without radical surgery treated with proton radiation.^23^ Moreover, Takayama et al. revealed that the 3‐year overall survival (OS), LC, and regional control (RC) rates for locally advanced oral cavity cancer treated with proton boost radiation were 87.0, 86.6, and 83.9%, respectively, demonstrating effective results.^22^ Nevertheless, clinical evidence linking PBRT for HNSCC with curative effectiveness and toxicity, particularly for carbon ions, is still insufficient given the variations in morbidity, radiosensitivity, and biological behavior of different primary sites.

We recently published preliminary findings regarding the benefit of PBRT in major salivary gland carcinomas, showing a 2‐year local‐regional recurrence‐free survival rate of 94.2% and mild toxicities.^16^ In the present study, we detailed the preliminary results of patients with HNSCC treated at the Shanghai Proton and Heavy Ion Center (SPHIC) using proton, carbon ion, or mixed‐beam RT employing proton or photon plus a carbon ion boost, providing clinical evidence for the efficacy of PBRT in HNSCC.

## 2 METHODS

### 2.1 Pretreatment evaluation

This retrospective research covered 68 consecutive patients treated at the SPHIC between August 2015 and December 2020 who had HNSCC (oral cavity, oropharynx, larynx, and hypopharynx) that had been newly diagnosed and histologically verified. Patients with distant metastases and prior head and neck radiotherapy for any malignancy were ineligible. As part of the pretreatment evaluation, all patients should have a history‐taking and physical assessment, as well as an enhanced magnetic resonance imaging (MRI) or computed tomography (CT) of the head and neck region. To rule out distant metastases, either positron emission tomography (PET)/CT was used, or a combination of chest CT, abdominal ultrasonography, and bone scanning. The American Joint Committee on Cancer (AJCC) staging system for this disease was used to stage all patients.

### 2.2 Radiotherapy

All patients had simulation CT scanning from the vertex to the inferior margin of the clavicular head after being positioned in supine position and fitted with thermoplastic masks to immobilize their heads, necks, and shoulders. Then, to identify targets and organs at risks (OARs), simulated CT was combined with the MRI used in the treatment position. The primary lesion and metastatic lymph nodes identified on clinical examination or radiographic evidence were contoured in the gross tumor volume (GTV). The GTV with a 1–3 mm margin to achieve the prescribed dose was designated as the clinical target volume (CTV) for boost (CTV‐boost). The high‐risk clinical target volume (CTV1) includes the CTV‐boost (or tumor bed) and subclinical extension according to clinical high‐risk area. In the case of individuals with ipsilateral upper neck lymphadenopathy, nodal levels IV and Vb were contained in CTV2. Based on the CTV, the planning target volume was formed by adding 3 mm for setup variability and 3–5 mm for range uncertainty.

The total dose coverage of the targets and the OAR limitations were integrated and analyzed as a sum plan after the physical planning of a mixed photon or proton and carbon ion boost was evaluated individually. Except for the temporal lobes, brain stem, spinal cord, and optic nerve, which were based on previous experience from the National Institute of Quantum and Radiation Science (NIQRS) of Japan, the dosage limitations of the OARs are based on TD5/5 as stated by Emami et al.^24^, ^25^ Planning specifics for head and neck PBRT have already been addressed in depth.^26^ Five to seven‐field technique or double‐ or triple‐arc volumetric‐modulated arc therapy was applied for IMRT plans. The radiotherapy regimen of the patient is made according to the intent of RT (adjuvant or radical), the status of surgery and other factors, as well as the physician's experience.

### 2.3 Systemic therapy

Patients were advised to receive concurrent chemotherapy, which consisted of weekly or tri‐weekly doses of either cisplatin or nedaplatin, if their disease was locally advanced (i.e., T3), node positive, and/or had high‐risk pathological factors after surgery, such as positive margin and extracapsular extension of lymph node metastasis. In general, adjuvant chemotherapy was not offered.

### 2.4 Follow‐up and toxicity evaluation

After the termination of radiotherapy, all patients followed the same routine for follow‐up appointments. Four to 6 weeks after the end of RT, the first follow‐up visit was planned. Thereafter, visits were set for every 3 months for the first 2 years, every 6 months for the following 3 years, and then once a year thereafter. The Radiation Therapy Oncology Group (RTOG) was used to grade late adverse events (occurring >3 months after initiation of RT), and the toxicity criteria of the Common Terminology Criteria for Adverse Events (CTCAE 4.0) was used to grade acute toxicities (occurring within 3 months after initiation of RT).

### 2.5 Statistics

From the date of the pathology diagnosis to the date of death or the last follow‐up, the time of survival was determined. The interval between the date of diagnosis and documented failure date was used to calculate the time to local, regional, or distant failure. The Kaplan–Meier method was used to calculate survival rates, and the Cox proportional hazard analysis approach was used to identify independent prognostic factors. The statistical program SPSS (version 23.0) was used for all analyses.

## 3 RESULTS

### 3.1 Patient characteristics and treatment modality

Sixty‐eight consecutive newly diagnosed HNSCC patients who underwent proton, carbon ion, mixed photon, or proton and carbon ion boost at the SPHIC between August 2015 and December 2020 were examined. For the entire group, the median follow‐up was 24.5 months (range, 3–65). The oral cavity (*n* = 29), oropharynx (*n* = 22), hypopharynx (*n* = 10), and larynx (*n* = 7) were the primary tumor locations. The T category/N category (T3‐T4/N2‐3) or a positive surgical margin were indicators of adjuvant radiation in 27 patients who underwent prior surgery with R0 or R1 resection. Proton alone (*n* = 21), carbon ion alone (*n* = 10), proton combined with carbon ion boost (*n* = 24), and photon combined with carbon ion boost (*n* = 13) were administered to all patients. Twenty‐seven patients who underwent R0/R1 resection received adjuvant proton RT (56–66 GyE/28–33 fractions, *n* = 17) or adjuvant carbon ion RT (54–63 GyE/18–21 fractions, *n* = 10). Thirty‐seven patients with R2 resection or unresectable disease received proton (56 GyE/28 fractions) combined with carbon ion boost (12–18 Gy/4–6 fractions) in 24 cases and photon (56 Gy/28 fractions) combined with carbon ion boost (15–18 Gy/5–6 fractions) in 13 cases (*n* = 13), all with curative intent. The remaining four patients with unresectable disease received radical proton RT (70 GyE/35 fractions, *n* = 4). Table 1 lists the baseline and treatment characteristics of the 68 patients included in this study.

**TABLE 1:** Characteristics of the patients, their disease, and treatments.

Characteristic	No. of patients (%)
Median age (range)	56 (26–83)
Gender	
Male	51 (75)
Female	17 (25)
Primary site	
Oral cavity	29 (42.6)
Oropharynx	22 (32.4)
Hypopharynx	10 (14.7)
Larynx	7 (10.3)
Surgery status	
R0	18 (26.5)
R1	9 (13.2)
R2	5 (7.4)
No surgery	36 (52.9)
RT parameter	
Adjuvant RT	27 (39.7)
Radical RT	41 (60.3)
Clinical T category	
T1	14 (20.6)
T2	22 (32.4)
T3	12 (17.6)
T4	20 (29.4)
Clinical N category	
N0	20 (29.4)
N1	19 (27.9)
N2	26 (38.3)
N3	3 (4.4)
Stage	
I	11 (16.2)
II	5 (7.3)
III	14 (20.6)
IV	38 (55.9)
Radiotherapy technique	
IMPT	21 (30.9)
IMCT	10 (14.7)
IMRT+IMCT	13 (19.1)
IMPT+IMCT	24 (35.3)
PEG before RT	
With	18 (26.5)
Without	50 (73.5)
Induction chemotherapy	
With	21 (30.9)
Without	47 (69.1)
Concurrent chemotherapy	
With	25 (36.8)
Without	43 (63.2)

Abbreviations: IMCT, intensity‐modulated carbon‐ion radiotherapy; IMPT, intensity‐modulated proton radiotherapy; PEG, percutaneous gastroectomy; RT, radiotherapy.

### 3.2 Survival

**FIGURE 1:**
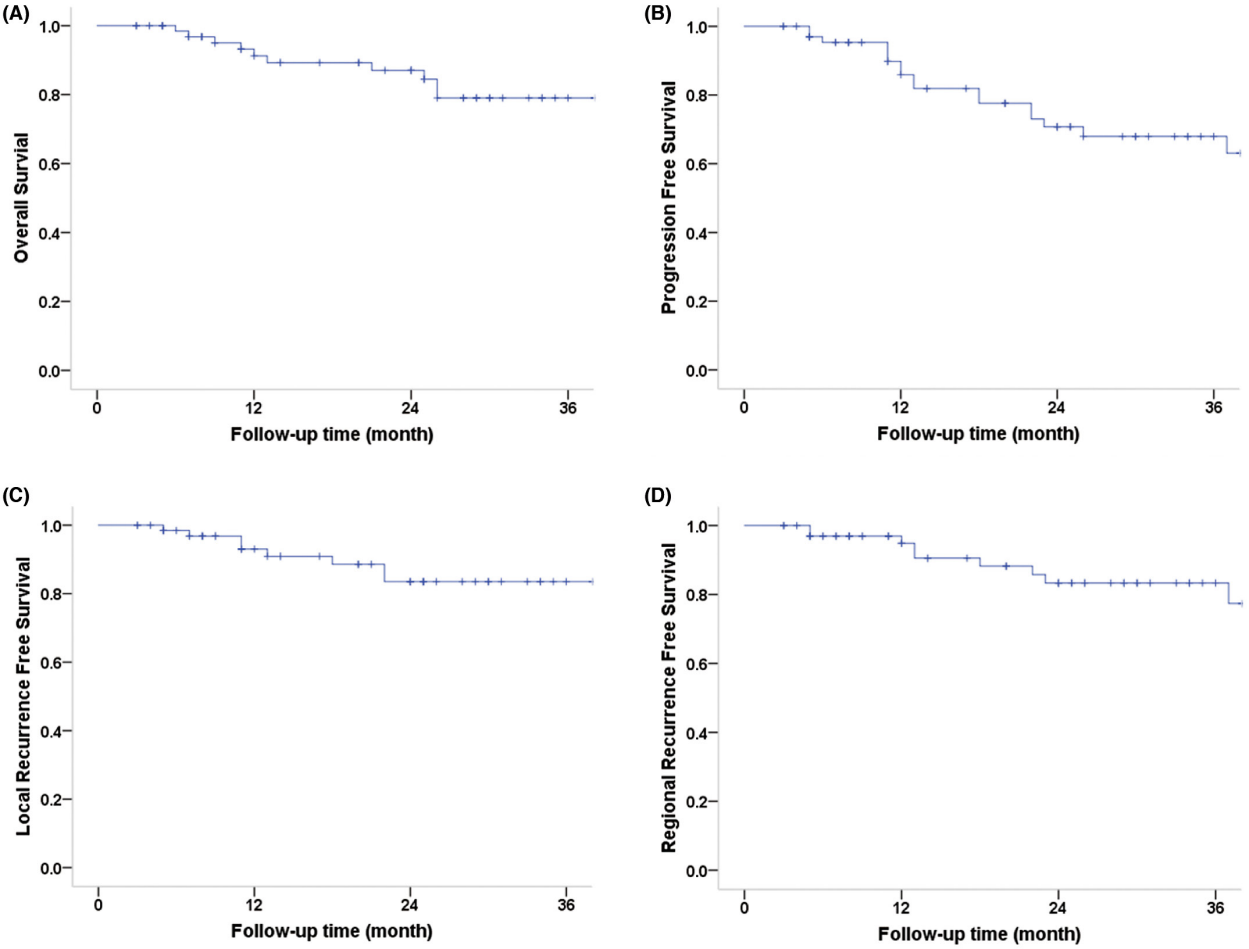
Overall survival (OS), progression‐free survival (PFS), local recurrence‐free survival (LRFS), and regional recurrence‐free survival (RRFS) curves of the entire cohort. The 3‐year OS, PFS, LRFS, and RRFS rates were 79.0%, 67.9%, 83.5%, and 83.3%, respectively.

After a median follow‐up of 24.5 months (range, 3–65), seven patients died from local and/or regional recurrence, one patient died from incision infection after mucosal necrotic excision, and another two patients died from other diseases (such as diabetes). Sixteen patients experienced recurrence events: six with local recurrence only, six with regional failure only, and four with both local and regional recurrences. In addition, three patients developed distant metastases (lung or bone metastases). The 3‐year OS, disease‐specific survival (DSS), progression‐free survival (PFS), local recurrence‐free survival (LRFS), regional recurrence‐free survival (RRFS), and distant metastasis‐free survival (DMFS) rates were 79.0%, 84.7%, 67.9%, 83.5%, 83.3%, and 96.1%, respectively, for the entire cohort (Figure 1A–D).

### 3.3 Predictive factors for OS, PFS, LRFS, and RRFS

**FIGURE 2:**
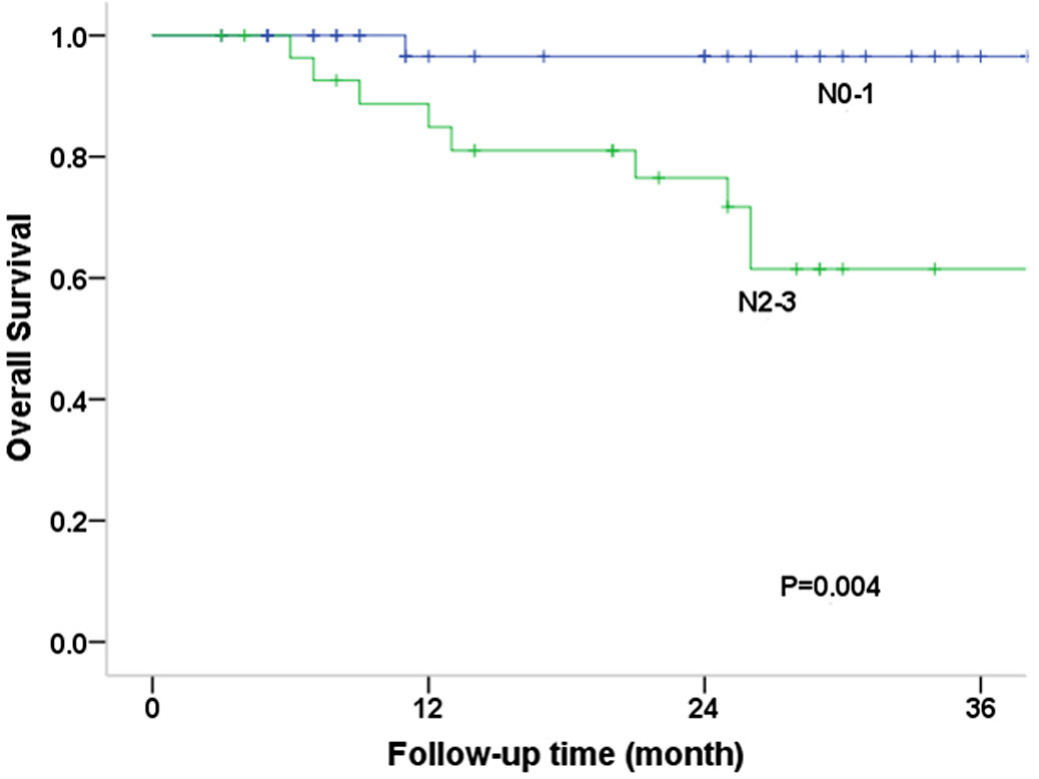
Curves of overall survival (OS) stratified by N category (N0‐1 versus N2‐3), and the corresponding 3‐year OS rates were 96.6% for patients with N0‐1 disease, and 61.5% for patients with N2‐3 disease.

**FIGURE 3:**
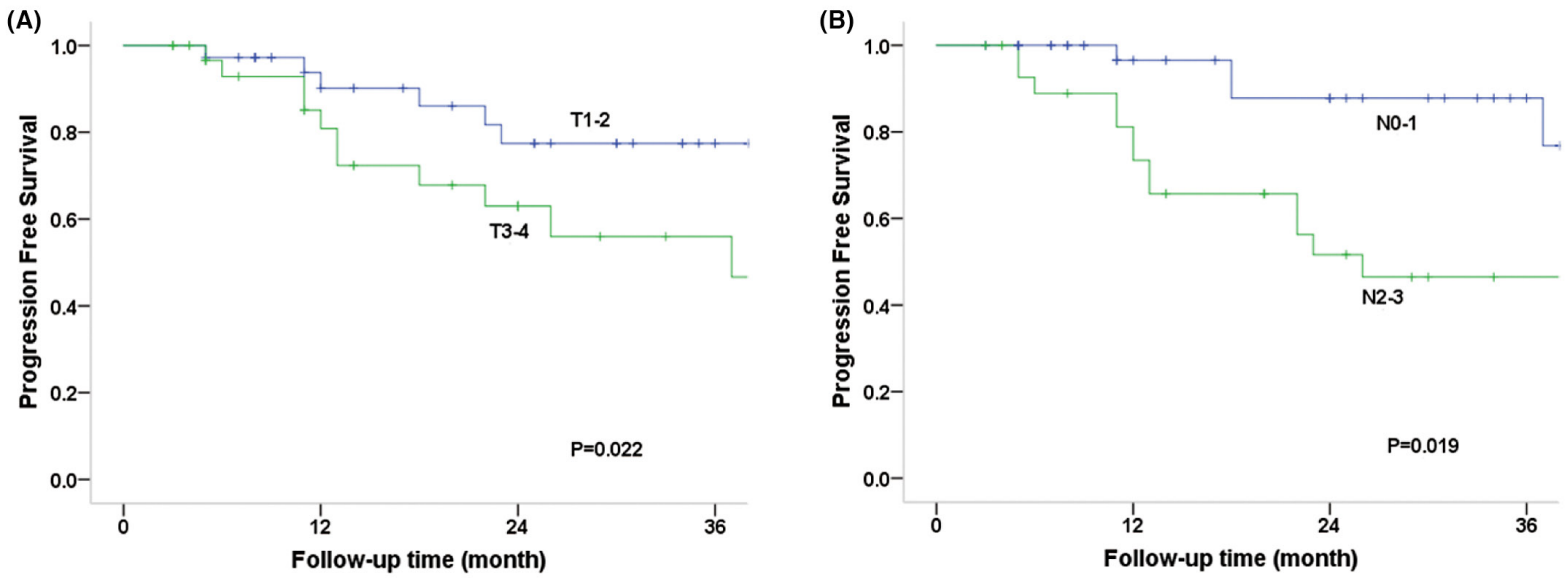
Progression‐free survival (PFS) curves according to T and N category. The 3‐year PFS rates were 77.4% and 56.0% for T1–2 versus T3/4 disease, and 87.8% and 46.5% for N0‐1 versus N2‐3, respectively.

**FIGURE 4:**
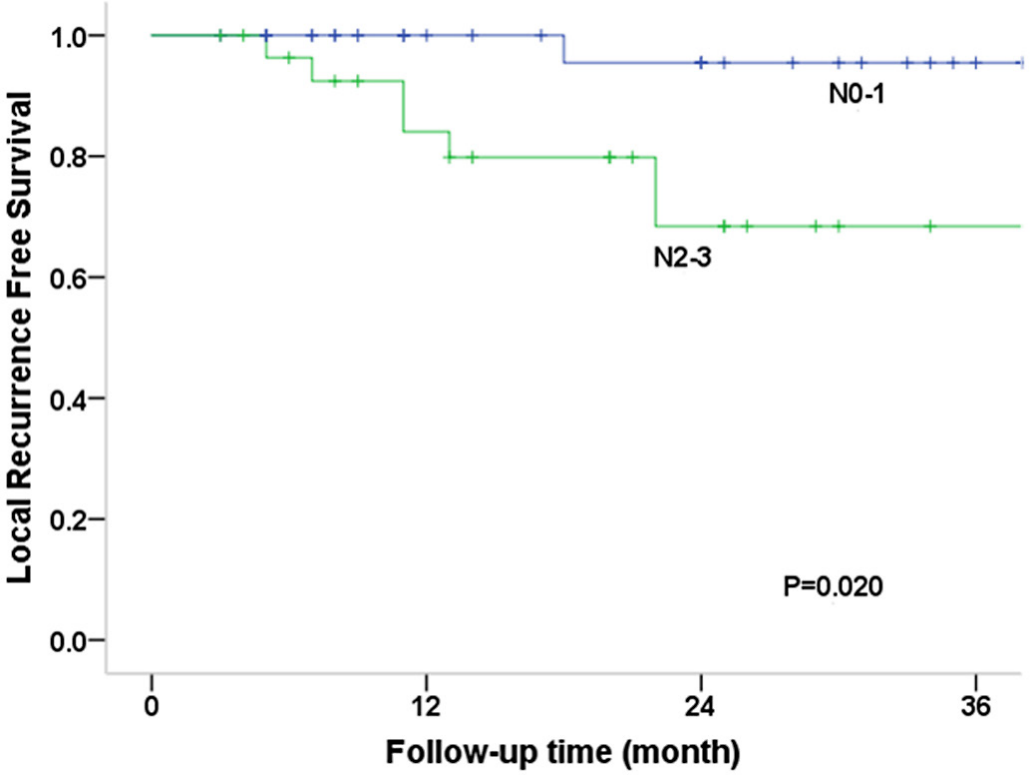
Local‐regional recurrence‐free survival (LRFS) curves according to N category. The 3‐year LRFS rates were 95.5% and 68.4% for N0–1 and N2–3 disease, respectively.

**FIGURE 5:**
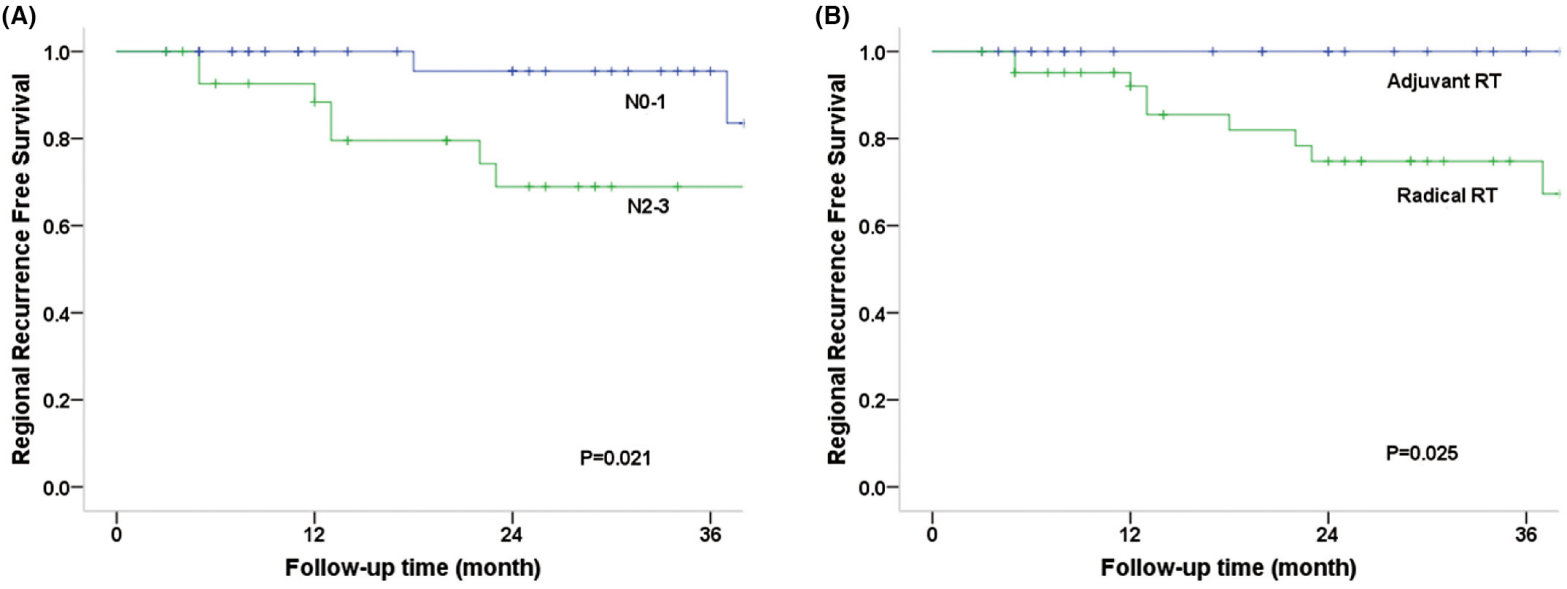
Regional recurrence‐free survival (RRFS) curves according to N category and RT parameters. The 3‐year RRFS rates were 100% for cases with adjuvant radiotherapy and 74.8% for definitive RT, and 95.5% for N0–1 and 68.9% for N2–3, respectively.

The log‐rank test was used to compare survival curves for factors such as age (56 vs. >56 years), sex (male vs. female), primary site, RT parameter (adjuvant vs. radical RT), stage (I–II vs. III–IV), induction chemotherapy, and concurrent chemotherapy status (Table 2). OS was substantially correlated with N categorization according to univariate analysis (*p* = 0.004), and the 3‐year OS rates for patients with N0–1 and N2–3 disease were 96.6% and 61.5%, respectively (Figure 2). Sex (*p* = 0.018), primary site (*p* = 0.029), T category (*p* = 0.022), and N category (*p* = 0.019) were significantly correlated with PFS. For T1–2 versus T3/4 disease, the 3‐year PFS rates were 77.4% and 56.0%, respectively, and for N0–1 versus N2–3, they were 87.8% and 46.5%, respectively (Figure 3A,B). Additionally, the N2–3 category was significantly associated with an increased risk of local failure (*p* = 0.020), comprising 68.4% of LRFS cases (Figure 4). Additionally, the findings indicated statistical differences in the N category (*p* = 0.021), RT parameter (*p* = 0.025), and sex (*p* = 0.039) for RRFS. The 3‐year RRFS rates were 100% in patients treated with adjuvant RT, 74.8% in cases treated with definitive RT, 95.5% in cases of N0–1, and 68.9% in cases of N2–3, respectively (Figure 5A,B). The outcomes of the multivariate analysis showed that N category was a substantial independent prognostic factor of OS (*p* = 0.02), PFS (*p* = 0.04), and RRFS (*p* = 0.01), that sex was a significant predictor of PFS (*p* = 0.02), and that age was a prognostic factor for RRFS (*p* = 0.03) (Table 3).

**TABLE 2:** Univariate analysis for the 3‐year OS, PFS, LRFS, RRFS, and DMFS.

Factor	3y OS	*p* Value	3y PFS	*p* Value	3y LRFS	*p* Value	3y RRFS	*p* Value	3y DMFS	*p* Value
Age		0.749		0.915		0.450		0.195		0.437
≤56	0.778		0.602		0.754		0.863		0.957	
>56	0.791		0.755		0.926		0.793		0.964	
Gender		0.065		**0.018**		0.175		**0.039**		0.286
Male	0.726		0.605		0.805		0.777		0.947	
Female	1.0		0.917		0.917		1.0		1.0	
Primary site		**0.054**		**0.029**		0.383		0.770		**0.023**
Oral cavity	0.779		0.691		0.792		0.838		0.966	
Oropharynx	0.917		0.746		0.917		0.746		1.0	
Hypopharynx	0.450		0.313		0.700		0.833		0.800	
Larynx	1.0		1.0		1.0		1.0		1.0	
RT parameter		0.328		0.069		0.388		**0.025**		0.228
Radical	0.753		0.602		0.813		0.748		0.938	
Adjuvant	0.861		0.834		0.872		1.0		1.0	
T category		0.255		**0.022**		0.154		0.175		0.522
T1–2	0.843		0.774		0.891		0.882		0.972	
T3–4	0.732		0.560		0.768		0.776		0.947	
N category		**0.004**		**0.019**		**0.020**		**0.021**		0.628
N0–1	0.966		0.878		0.955		0.955		0.955	
N2–3	0.615		0.465		0.684		0.689		0.963	
Stage		0.1		0.144		0.140		0.577		0.466
I–II	1.0		0.857		1.000		0.857		1.000	
III–IV	0.741		0.635		0.796		0.823		0.951	
Induction chemotherapy		0.891		0.646		0.833		0.603		0.786
With	0.768		0.535		0.820		0.756		0.933	
Without	0.811		0.768		0.842		0.880		0.977	
Concurrent chemotherapy		0.360		0.519		0.912		0.517		0.909
With	0.698		0.520		0.824		0.737		0.929	
Without	0.841		0.765		0.835		0.881		0.976	

Abbreviations: DMFS, distant metastasis‐free survival; LRRFS, local‐regional recurrence‐free survival; OS, overall survival; PFS, progression‐free survival. Statistically significant differences (*p* ≤ 0.05) indicated in bold type.

**TABLE 3:** Cox proportional hazard regression analysis for 3‐year OS, PFS, LRFS, and RRFS.

Factors	OS		PFS		LRFS		RRFS	
	*p*	HR (95% CI)	*p*	HR (95% CI)	*p*	HR (95% CI)	*p*	HR (95% CI)
Age	‐	‐	‐	‐	0.84	1.20 (0.20–7.21)	**0.03**	6.00 (1.20–29.94)
Gender	‐	‐	**0.02**	0.09 (0.01–0.69)	0.14	0.17 (0.02–1.79)	‐	‐
RT parameters	0.72	0.71 (0.11–4.56)	0.29	2.11 (0.53–8.47)	0.74	1.36 (0.22–8.55)	‐	‐
Primary site	0.31	1.53 (0.68–3.48)	0.62	0.86 (0.47–1.57)	0.42	0.67 (0.25–1.77)	‐	‐
T category	0.56	0.66 (0.16–2.70)	**0.07**	0.38 (0.14–1.08)	0.21	0.38 (0.08–1.73)	0.85	0.86 (0.18–4.16)
N category	**0.02**	0.07 (0.01–0.69)	**0.04**	0.35 (0.13–0.94)	0.09	0.19 (0.03–1.33)	**0.01**	0.10 (0.02–0.56)

Abbreviations: CI, confidence interval; DMFS, distant metastasis‐free survival; PFS, progression‐free survival. Statistically significant differences (*p* ≤ 0.05) indicated in bold type.

### 3.4 Toxicities

The most frequently reported acute adverse effects were mucositis, dermatitis, xerostomia, and dysphagia. Two patients presented with Grade 3 mucositis and dysphagia. Thirty‐six (53%), 14 (21%), 14 (21%), and 6 (9%) patients developed Grade 2 mucositis, dermatitis, xerostomia, and dysphagia, respectively. The incidence of weight loss during RT was 35% (25% Grade 1 and 10% Grade 2). Gastrostomy was performed in 18 patients (26.5%) before RT, which may affect the grade of mucositis based on CTCAE criteria, while acute mucositis was graded using the RTOG criteria, with 31 cases (45.6%) of Grade 3 and 28 (41.2%) of Grade 2.

Additionally, the treatment exacerbated long‐term side effects, such as dysphagia, xerostomia, and mucosal necrosis. Two patients (2%) each experienced Grade 4 and Grade 3 mucosal necrosis. Thirteen (19%) and 8 (12%) patients had Grade 2 xerostomia and dysphagia, respectively. RT‐related acute and late toxicities are shown in Table 4.

**TABLE 4:** Type and frequency of adverse events.

Toxicity	Grade, *n* (%)				
	1	2	3	4	5
Acute					
Mucositis	31 (46)	36 (53)	1 (1)	0	0
Dermatitis	49 (72)	14 (21)	0	0	0
Xerostomia	37 (54)	14 (21)	0	0	0
Weight loss	17 (25)	7 (10)	0	0	0
Dysphagia	8 (12)	6 (9)	1 (1)	0	0
Dysgeusia	16 (24)	0	0	0	0
Late					
Xerostomia	48 (71)	13 (19%)	0	0	0
Dysphagia	5 (7)	8 (12%)	0	0	0
Dysgeusia	18 (26)	1 (1%)			
Necrosis	—	—	1 (1)	1 (1)	0
Hearing impairment	3 (4)	2 (3%)	0	0	0

## 4 DISCUSSION

The results of 68 consecutive, non‐selected patients with HNSCC who received adjuvant or definitive RT at the SPHIC are presented in this study. The 3‐year OS, DSS, PFS, LRFS, RRFS, and DMFS rates were 79.0%, 84.7%, 67.9%, 83.5%, 83.3%, and 96.1%, respectively, with a median follow‐up length of 24.5 months (range, 3–65). Local, regional, and distant failure (lung or bone metastases) occurred in six, six, and three patients, respectively. Both local and regional recurrences occurred in the other four cases. In terms of acute toxicities, two patients demonstrated severe mucositis or dysphagia, and two patients also displayed late toxicities with significant mucosal necrosis.

In a study on the clinical outcomes of patients with locally advanced HNSCC treated with photon‐based chemoradiotherapy, Leeman et al. found that the 5‐year OS rates for herpes virus (HPV)‐associated oropharyngeal carcinoma (OPC), HPV‐negative OPC, oral cavity carcinoma, laryngeal carcinoma, and hypopharyngeal carcinoma were 85.2%, 66.9%, 53.2%, 56.3%, and 48.9%, respectively.^7^ Compared to the low linear energy transfer of photons, protons or carbon ions have higher relative biological effectiveness, which likely contributes to the improved clinical effects.^27^, ^28^ Takayama et al. reported the results of chemoradiotherapy (photon: 36 Gy/20 fractions) followed by proton beam boost (28.6–39.6 GyE/13–18 fractions) for locally advanced oral cavity carcinoma, with 3‐year OS, LC, and RC rates of 87.0, 86.6, and 83.9%, respectively, showing satisfactory outcomes.^22^ Furthermore, Kitabatake et al. also found that in elderly patients with difficulty undergoing surgery, the 3‐year LC and OS rates were 69% and 56%, respectively, for locally advanced oral carcinoma treated with proton irradiation.^23^ In addition, Aljabab et al. showed a 2‐year actuarial OS and PFS of 95.7% and 93.5%, respectively, in 46 patients with locally advanced OPC treated with intensity‐modulated proton therapy,^29^ and similarly good results of OPC with proton therapy were achieved in Gunn et al.'s study, with a 2‐year OS of 94.5%.^10^ However, in a case‐matched study between protons and photons for OPC, no statistically significant differences in survival benefits were shown.^20^ In fact, a comparative study of proton versus photon therapy for the survival benefits of head and neck cancers is scarce, most of which focuses on side effects. In the present study, the majority of patients who received proton RT underwent R0 or R1 resection. The 3‐year OS, LRFS, and RRFS rates of patients who received adjuvant therapy were 86.1%, 87.2%, and 100%, respectively, indicating that the use of PBRT as postoperative adjuvant radiation may be appropriate.

A preclinical investigation discovered that carbon‐ion beam treatment was more beneficial to HPV‐negative cell lines and might be an effective HNSCC treatment option.^30^ The 5‐year LC rate was 61% for patients with HNSCC, according to Mizoe et al.'s evaluation of the effects of carbon‐ion radiation for head and neck cancer.^31^ As demonstrated by Koto et al.^32^ the 1‐ and 3‐year LC and OS rates of locally advanced SCC of the external auditory canal and middle ear treated by carbon ion RT were 72% and 54%, and 70% and 40%, respectively, while the prognosis of patients with corresponding diseases who underwent photon RT remains poor, with a 2‐year OS of only 22%,^33^ indicating that specific types of HNSCC with general radioresistance may be overcome by the use of a carbon ion beam. In a phase II trial conducted by Hauswald et al. to assess the feasibility and safety of a therapy regimen based on carbon ion boost (24 Gy [relative biological effectiveness] in 8 fractions) and IMRT (50 Gy in 25 fractions) in locally advanced HNSCC, it was reported that the 1‐year OS, PFS, and LC rates were 100.0%. Despite the small sample size of only eight patients, the results of this mixed‐particle RT treatment were encouraging.^21^ Currently, clinical studies on the use of particle therapy, especially for carbon ions, for newly diagnosed HNSCC are limited. In the present study, 69.1% of the patients received carbon‐based radiation therapy, and the 3‐year OS, PFS, LRFS, and RRFS rates for the entire cohort were 79.0%, 67.9%, 83.5%, and 83.3%, respectively. It should also be noted that most (76.5%) cases analyzed were locally advanced stages. Therefore, the effectiveness of PBRT in treating HNSCC suggested by our findings is encouraging. We found that of the 10 patients who died during follow‐up, seven died of disease progression, whereas the remainder died of non‐tumor‐related causes, thus affecting OS for the entire cohort. The 3‐year DSS rate was 84.7% when only death due to HNSCC was considered, which was satisfactory.

In term of toxicities, patients with HNSCC treated with proton therapy have been reported to present with a lower incidence of toxicities, including mucositis, xerostomia, dysgeusia, tube feeding, and weight loss.^19^, ^34^, ^35^ In a study by Blanchard et al., a case‐matched analysis was performed to assess the adverse effects of protons versus photons on OPC.^20^ At 3 months and 1 year after RT, feeding tube reliance and Grade 3 weight loss were 18% and 34% (*p* = 0.05) and 8% versus 24.7% (*p* = 0.01), respectively. Manzar et al. also explored whether this physical superiority of protons for OPC could translate into clinical benefits compared to photons and the decrease in the incidence of Grade 3 mucositis (14.7% vs. 35.9%) and Grade 2 pain (3.2% vs. 24.3%) in the proton group.^36^ Similar results were reported by Romesser et al. with significantly less Grade 2 or greater acute dysgeusia (5.6% vs. 65.2%) and mucositis (16.7% vs. 52.2%) with proton beam RT.^37^ In addition, Zhang et al. examined the incidence of osteoradionecrosis and mandibular dosage following proton or photon irradiation. The proton group had lower mean mandibular doses (25.6 vs. 41.2 Gy) and lower osteoradionecrosis rates (proton group: 2% [1 case with Grade 1]; photon group: 7.7% [23 cases with Grade 1, 1 case with grade 2, 5 cases with Grade 3, and 12 cases with Grade 4]).^38^ The present study did not report RT‐related osteoradionecrosis after PBRT. In addition to the therapeutic effect, the toxicities induced by carbon‐ion boost combined with photons were also assessed in a study by Hauswald et al. of four patients (50%) with Grade 3 mucositis wherein only one case with Grade 3 dysphagia occurred.^21^

Results regarding toxicities induced by mixed treatment based on carbon ions for HNSCC are limited. Hu et al. found that mixed photons with carbon ion boost were well tolerated in nasopharyngeal cancer patients, with only two patients experiencing significant acute toxicities (Grade 3 dermatitis).^39^ Previous research concerning the use of mixed PBRT in major salivary gland cancers found only one patient with Grade 3 mucositis.^16^ In our study, the treatment of photons or protons combined with a carbon ion boost was also employed with radical RT intent. Except for one patient with Grade 3 mucositis and one with Grade 3 dysphagia, most patients experienced mild to moderate acute side effects. As the grade of mucositis based on CTCAE criteria may also have been affected by the fact that 18 patients underwent gastrostomy prior to RT for their nutritional status during treatment, RTOG criteria were utilized to grade acute mucositis as well, with 46% of cases manifesting acute Grade 3 mucositis. The above studies demonstrate that the superior physical properties of PBRT make it well tolerated in HNSCC treatment.

The current study has a few limitations that need to be addressed. First, the median follow‐up period (24.5 months) was quite brief; a longer follow‐up is required for tumors with specific subsites or varied HPV status to assess long‐term outcomes and toxicities. Second, the effectiveness and adverse effects of treatment may be affected by patient‐specific variations in RT techniques. Further stratified analysis based on various RT modalities needs to be performed. Third, there were relatively few patients included in our investigation, which was a retrospective analysis. The majority of earlier research on this topic have also been constrained by their retrospective nature and sample sizes due to the heterogeneity among subsites and the rarity of HNSCC. However, the design and execution of a prospective clinical study is challenging.

## 5 CONCLUSION

In conclusion, our findings highlight that PBRT generates moderate acute and late toxicities and demonstrates promising treatment efficacy in patients with HNSCC. The relative 3‐year OS, DSS, PFS, LRFS, RRFS, and DMFS rates were 79.0%, 84.7%, 67.9%, 83.5%, 83.3%, and 96.1%, respectively. Long‐term follow‐up is required to confirm the benefits and safety of PBRT in HNSCC management. Prospective evaluation of clinical outcomes with PBRT for HNSCC is further warranted, with an emphasis on clinical effectiveness as well as side effects.

## AUTHOR CONTRIBUTIONS

**Qingting Huang:** Data curation (equal); funding acquisition (equal); investigation (equal); software (equal); validation (equal); writing – original draft (lead). **Jiyi Hu:** Conceptualization (equal); data curation (equal); software (equal); validation (equal); writing – review and editing (equal). **Weixu Hu:** Data curation (equal); formal analysis (equal); methodology (equal); software (equal). **Jing Gao:** Methodology (equal); project administration (equal); resources (equal); supervision (equal); visualization (equal). **Jing Yang:** Data curation (equal); formal analysis (equal); resources (equal). **Xianxin Qiu:** Data curation (equal); investigation (equal); resources (equal); software (equal). **Haojiong Zhang:** Formal analysis (equal); investigation (equal); methodology (equal); software (equal). **Jiade Jay Lu:** Conceptualization (equal); supervision (equal); validation (equal); visualization (equal); writing – review and editing (equal). **Lin Kong:** Conceptualization (lead); funding acquisition (equal); methodology (equal); resources (equal); validation (lead); writing – review and editing (lead).

## FUNDING INFORMATION

This work was mainly supported by the Key Research and Development Program of the Ministry of Science and Technology (project no. 2022YFC2401505), the Shanghai Sailing Program (project no. 22YF1444800), the Science and Technology Development Fund of Shanghai Pudong New Area (project no. PKJ2020‐Y53), and the Health Industry Clinical Research Project of Shanghai Municipal Health Commission (project no. 20224Y0062).

## CONFLICT OF INTEREST STATEMENT

The authors declare no conflicts of interest related to this study.

## ETHICAL APPROVAL AND CONSENT TO PARTICIPATE

The authors are accountable for all aspects of the work in ensuring that questions related to the accuracy or integrity of any part of the work are appropriately investigated and resolved. This study was approved by the institutional review board (IRB) of the Shanghai Proton and Heavy Ion Center, Shanghai, China (No. 230208EXP‐01).

## PATIENT CONSENT FOR PUBLICATION

Not applicable.

## Data Availability

The datasets generated and/or analyzed during the current study are available from the corresponding author on reasonable request.

## References

[1] Johnson DE , Burtness B , Leemans CR , Lui VWY , Bauman JE , Grandis JR . Head and neck squamous cell carcinoma. Nat Rev Dis Primers. 2020;6:92. 33243986 10.1038/s41572-020-00224-3 PMC7944998

[2] Marur S , Forastiere AA . Head and neck squamous cell carcinoma: update on epidemiology, diagnosis, and treatment. Mayo Clin Proc. 2016;91:386‐396. 26944243 10.1016/j.mayocp.2015.12.017

[3] Ferlay J , Colombet M , Soerjomataram I , et al. Estimating the global cancer incidence and mortality in 2018: GLOBOCAN sources and methods. Int J Cancer. 2019;144:1941‐1953. 30350310 10.1002/ijc.31937

[4] Schick U , Huguet F , Pointreau Y , Pradier O . Radiotherapy for head and neck squamous cell carcinoma: state of the art and future directions. Cancer Radiother. 2017;21:498‐504. 28864046 10.1016/j.canrad.2017.07.032

[5] Bur AM , Lin A , Weinstein GS . Adjuvant radiotherapy for early head and neck squamous cell carcinoma with perineural invasion: a systematic review. Head Neck. 2016;38(Suppl 1):E2350‐E2357. 26613965 10.1002/hed.24295

[6] Fridman E , Na'ara S , Agarwal J , et al. The role of adjuvant treatment in early‐stage oral cavity squamous cell carcinoma: an international collaborative study. Cancer. 2018;124:2948‐2955. 29757457 10.1002/cncr.31531 PMC6607430

[7] Leeman JE , Li JG , Pei X , et al. Patterns of treatment failure and postrecurrence outcomes among patients with locally advanced head and neck squamous cell carcinoma after chemoradiotherapy using modern radiation techniques. JAMA Oncol. 2017;3:1487‐1494. 28542679 10.1001/jamaoncol.2017.0973 PMC5710194

[8] Kodaira T , Nishimura Y , Kagami Y , et al. Definitive radiotherapy for head and neck squamous cell carcinoma: update and perspectives on the basis of EBM. Jpn J Clin Oncol. 2015;45:235‐243. 25492926 10.1093/jjco/hyu209

[9] Setton J , Caria N , Romanyshyn J , et al. Intensity‐modulated radiotherapy in the treatment of oropharyngeal cancer: an update of the Memorial Sloan‐Kettering Cancer Center experience. Int J Radiat Oncol Biol Phys. 2012;82:291‐298. 21167652 10.1016/j.ijrobp.2010.10.041

[10] Gunn GB , Blanchard P , Garden AS , et al. Clinical outcomes and patterns of disease recurrence after intensity modulated proton therapy for oropharyngeal squamous carcinoma. Int J Radiat Oncol Biol Phys. 2016;95:360‐367. 27084653 10.1016/j.ijrobp.2016.02.021 PMC5474303

[11] Kraaijenga SA , Oskam IM , van Son RJ , et al. Assessment of voice, speech, and related quality of life in advanced head and neck cancer patients 10‐years+ after chemoradiotherapy. Oral Oncol. 2016;55:24‐30. 26874554 10.1016/j.oraloncology.2016.02.001

[12] Quinten C , Coens C , Mauer M , et al. Baseline quality of life as a prognostic indicator of survival: a meta‐analysis of individual patient data from EORTC clinical trials. Lancet Oncol. 2009;10:865‐871. 19695956 10.1016/S1470-2045(09)70200-1

[13] Ediebah DE , Quinten C , Coens C , et al. Quality of life as a prognostic indicator of survival: a pooled analysis of individual patient data from Canadian cancer trials group clinical trials. Cancer. 2018;124:3409‐3416. 29905936 10.1002/cncr.31556

[14] Leeman JE , Romesser PB , Zhou Y , et al. Proton therapy for head and neck cancer: expanding the therapeutic window. Lancet Oncol. 2017;18:e254‐e265. 28456587 10.1016/S1470-2045(17)30179-1

[15] Beddok A , Vela A , Calugaru V , et al. Proton therapy for head and neck squamous cell carcinomas: a review of the physical and clinical challenges. Radiother Oncol. 2020;147:30‐39. 32224315 10.1016/j.radonc.2020.03.006

[16] Huang Q , Hu W , Hu J , et al. Intensity‐modulated proton and carbon‐ion radiation therapy in the management of major salivary gland carcinomas. Ann Transl Med. 2022;10:1195. 36544665 10.21037/atm-20-7988 PMC9761122

[17] Cozzi L , Fogliata A , Lomax A , Bolsi A . A treatment planning comparison of 3D conformal therapy, intensity modulated photon therapy and proton therapy for treatment of advanced head and neck tumours. Radiother Oncol. 2001;61:287‐297. 11730999 10.1016/s0167-8140(01)00403-0

[18] Eekers DBP , Roelofs E , Jelen U , et al. Benefit of particle therapy in re‐irradiation of head and neck patients. Results of a multicentric in silico ROCOCO trial. Radiother Oncol. 2016;121:387‐394. 27639891 10.1016/j.radonc.2016.08.020

[19] Bagley AF , Ye R , Garden AS , et al. Xerostomia‐related quality of life for patients with oropharyngeal carcinoma treated with proton therapy. Radiother Oncol. 2020;142:133‐139. 31431373 10.1016/j.radonc.2019.07.012 PMC8457365

[20] Blanchard P , Garden AS , Gunn GB , et al. Intensity‐modulated proton beam therapy (IMPT) versus intensity‐modulated photon therapy (IMRT) for patients with oropharynx cancer – a case matched analysis. Radiother Oncol. 2016;120:48‐55. 27342249 10.1016/j.radonc.2016.05.022 PMC5474304

[21] Hauswald H , Jensen AD , Krauss J , et al. Phase II study of induction chemotherapy with docetaxel, cisplatin, 5‐fluorouracil followed by radioimmunotherapy with cetuximab and intensity‐modulated radiotherapy in combination with a carbon ion boost for locally advanced tumors of the oro‐, hypopharynx and larynx. Clin Transl Radiat Oncol. 2018;13:64‐73. 30370340 10.1016/j.ctro.2018.09.005 PMC6199783

[22] Takayama K , Nakamura T , Takada A , et al. Treatment results of alternating chemoradiotherapy followed by proton beam therapy boost combined with intra‐arterial infusion chemotherapy for stage III‐IVB tongue cancer. J Cancer Res Clin Oncol. 2016;142:659‐667. 26521257 10.1007/s00432-015-2069-0 PMC11819421

[23] Kitabatake T , Takayama K , Tominaga T , et al. Treatment outcomes of proton beam therapy combined with retrograde intra‐arterial infusion chemotherapy for locally advanced oral cancer in the elderly. Int J Oral Maxillofac Surg. 2022;51:1264‐1272. 35125267 10.1016/j.ijom.2022.01.014

[24] Emami B , Lyman J , Brown A , et al. Tolerance of normal tissue to therapeutic irradiation. Int J Radiat Oncol Biol Phys. 1991;21:109‐122. 2032882 10.1016/0360-3016(91)90171-y

[25] Chen AM , Yoshizaki T , Velez MA , Mikaeilian AG , Hsu S , Cao M . Tolerance of the brachial plexus to high‐dose reirradiation. Int J Radiat Oncol Biol Phys. 2017;98:83‐90. 28587056 10.1016/j.ijrobp.2017.01.244

[26] Hu W , Hu J , Huang Q , et al. Particle beam radiation therapy for adenoid cystic carcinoma of the nasal cavity and paranasal sinuses. Front Oncol. 2020;10:572493. 33102230 10.3389/fonc.2020.572493 PMC7556111

[27] Jiang GL . Particle therapy for cancers: a new weapon in radiation therapy. Front Med. 2012;6:165‐172. 22573221 10.1007/s11684-012-0196-4

[28] Wang L , Fossati P , Paganetti H , et al. The biological basis for enhanced effects of proton radiation therapy relative to photon radiation therapy for head and neck squamous cell carcinoma. Int J Part Ther. 2021;8:3‐13. 34285931 10.14338/IJPT-20-00070.1 PMC8270087

[29] Aljabab S , Liu A , Wong T , Liao JJ , Laramore GE , Parvathaneni U . Proton therapy for locally advanced oropharyngeal cancer: initial clinical experience at the University of Washington. Int J Part Ther. 2020;6:1‐12. 10.14338/IJPT-19-00053.1 PMC7038913 32582809

[30] Osu N , Kobayashi D , Shirai K , et al. Relative biological effectiveness of carbon ions for head‐and‐neck squamous cell carcinomas according to human papillomavirus status. J Pers Med. 2020;10:10. 32722522 10.3390/jpm10030071 PMC7565683

[31] Mizoe JE , Hasegawa A , Jingu K , et al. Results of carbon ion radiotherapy for head and neck cancer. Radiother Oncol. 2012;103:32‐37. 22321201 10.1016/j.radonc.2011.12.013

[32] Koto M , Hasegawa A , Takagi R , et al. Carbon ion radiotherapy for locally advanced squamous cell carcinoma of the external auditory canal and middle ear. Head Neck. 2016;38:512‐516. 25352333 10.1002/hed.23905

[33] Xie B , Zhang T , Dai C . Survival outcomes of patients with temporal bone squamous cell carcinoma with different invasion patterns. Head Neck. 2015;37:188‐196. 24347537 10.1002/hed.23576

[34] Lester‐Coll NH , Margalit DN . Modeling the potential benefits of proton therapy for patients with oropharyngeal head and neck cancer. Int J Radiat Oncol Biol Phys. 2019;104:563‐566. 31162056 10.1016/j.ijrobp.2019.03.040

[35] Meijer TWH , Scandurra D , Langendijk JA . Reduced radiation‐induced toxicity by using proton therapy for the treatment of oropharyngeal cancer. Br J Radiol. 2020;93:20190955. 31971818 10.1259/bjr.20190955 PMC7066974

[36] Manzar GS , Lester SC , Routman DM , et al. Comparative analysis of acute toxicities and patient reported outcomes between intensity‐modulated proton therapy (IMPT) and volumetric modulated arc therapy (VMAT) for the treatment of oropharyngeal cancer. Radiother Oncol. 2020;147:64‐74. 32234612 10.1016/j.radonc.2020.03.010

[37] Romesser PB , Cahlon O , Scher E , et al. Proton beam radiation therapy results in significantly reduced toxicity compared with intensity‐modulated radiation therapy for head and neck tumors that require ipsilateral radiation. Radiother Oncol. 2016;118:286‐292. 26867969 10.1016/j.radonc.2015.12.008 PMC4980117

[38] Zhang W , Zhang X , Yang P , et al. Intensity‐modulated proton therapy and osteoradionecrosis in oropharyngeal cancer. Radiother Oncol. 2017;123:401‐405. 28549794 10.1016/j.radonc.2017.05.006 PMC5779856

[39] Hu J , Huang Q , Gao J , et al. Mixed photon and carbon‐ion beam radiotherapy in the management of non‐metastatic nasopharyngeal carcinoma. Front Oncol. 2021;11:653050. 34367954 10.3389/fonc.2021.653050 PMC8343069

